# Potential of the World Health Organization’s Skin NTDs App to Support and Improve the Detection of Skin-Related Neglected Tropical Diseases: Protocol for a Performance Evaluation and Feasibility Study in Senegal

**DOI:** 10.2196/69420

**Published:** 2025-09-19

**Authors:** Dior Sall, Dominik Jockers, Pauline Dioussé, Jonas Wachinger, Gilbert Batista, Jose Antonio Ruiz Postigo, Laurene Petitfour, Charlotte Robert, Bachir Mansour Diallo, Fulgence Abdou Faye, Yacine Dieng, Maresa Neuerer, Agbogbenkou Tevi Dela-Dem Lawson, Felicitas Schwermann, Carme Carrion, Louis Hyacinthe Zoubi, Papa Mamadou Diagne, Christa Kasang, Fatou Ndiaye Oumar Sy, Mahamath Cisse, Till Bärnighausen, Madoky Diop

**Affiliations:** 1 Faculty of Medicine Iba Der Thiam University of Thies Thies Senegal; 2 Heidelberg Institute of Global Health Faculty of Medicine and University Hospital Heidelberg University Heidelberg Germany; 3 Department of Economics University of Göttingen Göttingen Germany; 4 Action Damien Dakar Senegal; 5 Prevention, Treatment and Care Unit Department of Control of Neglected Tropical Diseases World Health Organization Geneva Switzerland; 6 Department of Internal Medicine Abdou Aziz Sy Hospital Tivaouane Senegal; 7 Department of Community Health University of Alioune Diop de Bambey Bambey Senegal; 8 German Leprosy and Tuberculosis Relief Association DAHW Dakar Senegal; 9 German Leprosy and Tuberculosis Relief Association DAHW Würzburg Germany; 10 School of Health Sciences Universitat de Girona Girona Spain; 11 eHealth Lab Research Group eHealth Center Universitat Oberta de Catalunya Barcelona Spain; 12 National Leprosy Control Program Dakar Senegal; 13 Association Senegalaise de lutte contre la lèpre et les MTN Dakar Senegal; 14 Harvard Center for Population and Development Studies Harvard University Boston, MA United States; 15 Africa Health Research Institute Somkhele South Africa

**Keywords:** cutaneous, skin health, diagnostic, artificial intelligence, AI, image recognition, mobile health, mHealth, performance evaluation, community, capacity building, zero leprosy, qualitative research, quantitative research, West Africa

## Abstract

**Background:**

The World Health Organization (WHO) roadmap aims to control, eliminate, or eradicate neglected tropical diseases (NTDs) by promoting innovation in prevention, diagnosis, and treatment. In this context, mobile health (mHealth) tools could play an important role in improving health care across the globe, including for skin-related NTDs. One such tool is the WHO Skin NTDs App (currently available in its beta version), which utilizes artificial intelligence (AI) algorithms to classify skin lesion images and offers diagnostic suggestions and management information to bolster early detection at primary care levels. However, to harness the full potential of this and similar mHealth tools, additional insights into their diagnostic performance and potential implementation avenues in settings with limited access to trained dermatologists are essential.

**Objective:**

The objective of our mixed methods study is to test the functionality, operability, and potential of the AI-supported diagnostic component of the WHO Skin NTDs App (beta version) to support the detection of skin NTDs and common skin conditions in Senegal.

**Methods:**

We are conducting a diagnostic accuracy study combined with a qualitative preimplementation usability exploration. For the quantitative component, we will collect and analyze approximately 800 skin lesion images from patients presenting to the dermatology unit at the Thiès regional hospital in Senegal. Each lesion will be independently assessed by the AI-based WHO Skin NTDs App and by a dermatologist who will provide a diagnosis serving as the reference standard. Performance metrics, including accuracy, sensitivity, specificity, precision, *F*_1_-score, and area under the receiver operating characteristic curve, will be calculated for each diagnostic category to evaluate the app’s ability to detect skin-related NTDs. In parallel, we will conduct semistructured in-depth interviews with a purposive sample of 70-80 stakeholders, including policymakers, health care workers, community leaders, dermatologists, and members of leprosy-affected communities. Interviews will explore perceptions of the app’s usability, acceptability, and potential barriers and facilitators to its adoption within Senegal’s health system. Thematic analysis will be used to interpret qualitative data. Findings will help inform the design of an app-based intervention to be piloted in future community-level studies.

**Results:**

We expect the results to provide detailed insights into the feasibility and potential of the WHO Skin NTDs App to support and improve the detection of skin NTDs and common skin conditions at the community level in Senegal. We started data collection in August 2024, with the first results expected to be available in 2025.

**Conclusions:**

Our study will assess the performance and potential use of the WHO Skin NTDs App to detect skin NTDs and common skin conditions in Senegal, outlining its potential role in supporting early diagnoses and enhancing public health responses.

**Trial Registration:**

German Clinical Trials Register DRKS00034297; https://drks.de/search/de/trial/DRKS00034297/details

**International Registered Report Identifier (IRRID):**

DERR1-10.2196/69420

## Introduction

### Background

Neglected tropical diseases (NTDs) are estimated to affect more than 1 billion people worldwide, mostly in low- and middle-income countries [1]. With the NTD burden disproportionately high in disadvantaged communities, NTDs are rarely a priority in national and international health agendas or in the development of novel diagnostic tools or treatments [2]. To catalyze efforts, the World Health Organization (WHO) in 2020 set global targets to control and end the burden of NTDs by 2030 [1].

Skin NTDs are a subgroup of NTDs that manifest in lesions on the skin and can be detected by visual screening [3]. However, trained health care professionals who can reliably detect and diagnose such conditions are rare in many of the most affected settings [4]. Technological advancements are now promising to bridge this gap, especially in remote populations with limited access to resources [5-7].

**Figure 1:**
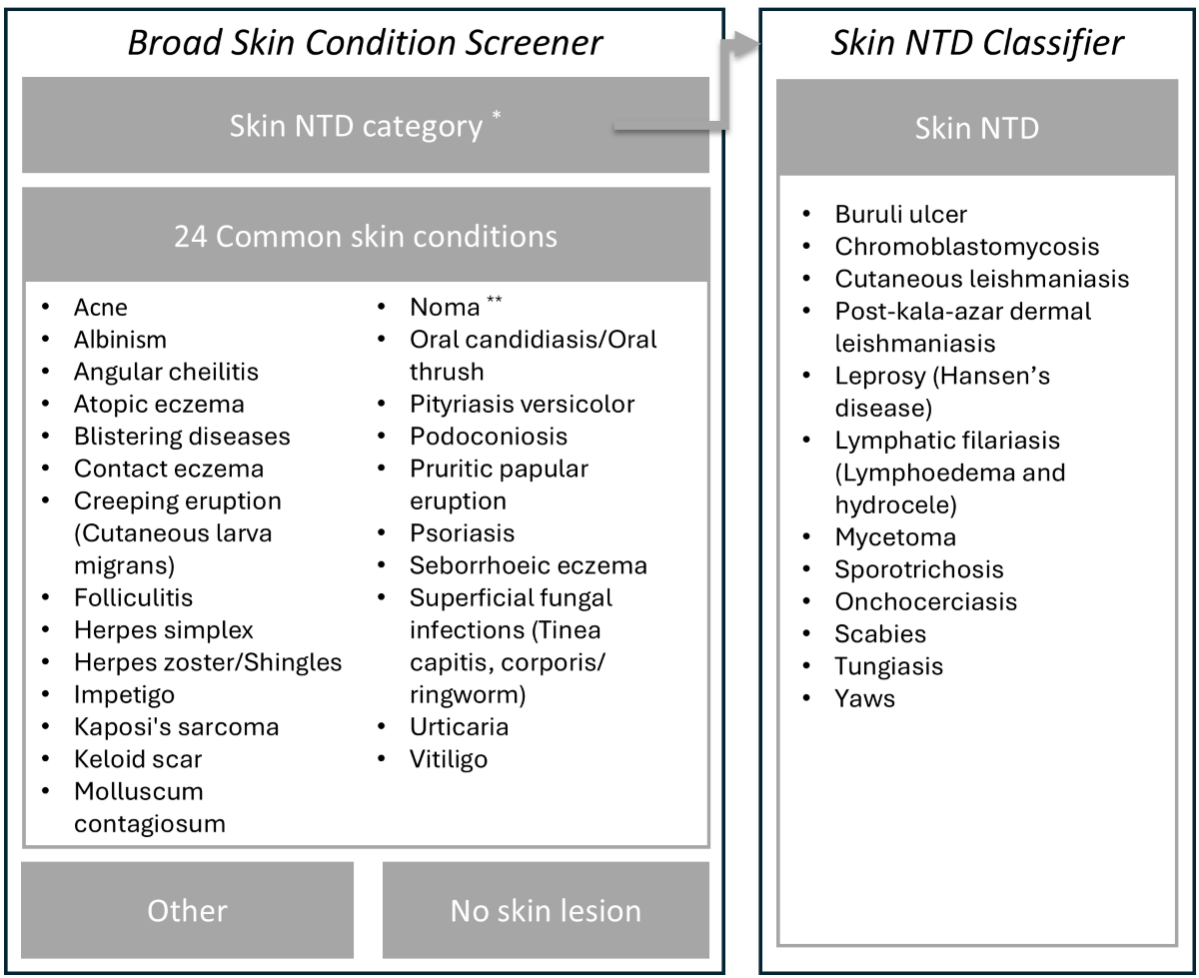
Coverage of algorithms included in the World Health Organization (WHO) Skin NTDs App (beta version). *The Broad Skin Condition Screener algorithm does not differentiate between the 12 skin NTDs included in this category. Instead, if this category is flagged, users are recommended to use the Skin NTD Classifier for specific diagnosis suggestion. **During app development, Noma was not considered a skin NTD. In December 2023, the WHO changed their assessment and included Noma on their list of NTDs. NTD: neglected tropical disease.

In 2023, the WHO integrated 2 artificial intelligence (AI) algorithms into their existing WHO Skin NTDs App. Using a convolutional neural network approach, the algorithms classify images to identify skin conditions. Integrating AI into the app aims at further supporting frontline health workers in efforts to detect skin NTDs and other skin conditions at early stages of manifestation [8]. The app allows users to take images of a skin lesion on their smartphone and provides them with a list of potential diagnoses. The app uses 2 AI algorithms in a cascade. The first algorithm (Broad Skin Condition Screener) classifies images across 24 common skin conditions, included as individual diagnoses, and one group covering all 12 skin NTDs. If a skin NTD is flagged, the second algorithm (Skin NTD Classifier) identifies which of the 12 NTDs is most likely present (see Figure 1) [9]. Although the use of the AI component requires internet connection, images can be stored offline and uploaded at a later stage. Beyond the AI algorithm, the app also features an offline diagnostic decision tree with branching logic to assess symptoms, and app users can access a general learning section on skin conditions. Previous research has outlined the user-friendliness of these offline features, as well as their potential role in frontline health worker capacity building [10].

The free-of-charge availability of the overarching Skin NTDs App presents a promising opportunity for large-scale implementation in resource-poor settings. However, the AI algorithm component so far has not been tested extensively in real-world applications [8]. Parallel to this study, the AI component is undergoing a first assessment in Kenya, with results expected before the end of 2025. Evaluations of other AI algorithm-based image analysis models for diagnosis have highlighted how performance differs across screened populations [11]. In general, variations in demographic characteristics, skin tones, and both the severity and complexity of skin conditions are likely to influence the detection accuracy [12]. Insights into potential avenues for implementing such algorithms in real-world detection efforts and for their integration into national programs are lacking [8].

To address these gaps, we will evaluate the performance of the WHO Skin NTDs App in the Senegalese setting. To assess the feasibility and acceptability of the app’s implementation, we are conducting qualitative interviews with stakeholders along the care cascade in Senegal. This is particularly critical for skin NTDs such as leprosy, where stigmatization of patients raises questions on the use of diagnostic tools at the community level [13,14]. Although early case detection might prevent disability and mitigate future stigma and psychological burden, maintaining diagnosis confidentiality is critical to protect patients.

### Study Objectives

The objective of our study is to investigate the potential of the AI component of the WHO Skin NTDs App (beta version) to support and improve the detection of skin NTDs and common skin diseases in Senegal. We will (1) evaluate the performance of the WHO Skin NTDs App algorithm in the detection of skin NTDs and common skin diseases and (2) qualitatively explore the feasibility and potential avenues for integrating the app into NTD detection processes at the community level. Our results will provide recommendations for the integration of this and similar tools into broader public health strategies to enhance the diagnosis and management of skin NTDs in Senegal and beyond.

## Methods

### Study Design

This study will combine quantitative and qualitative methods to evaluate the performance and potential of the novel AI diagnostic component of the WHO Skin NTDs App in the Senegalese context. This study protocol has been developed in accordance with the SPIRIT (Standard Protocol Items: Recommendations for Interventional Trials) guidelines (Multimedia Appendix 1).

The quantitative component will evaluate the performance of the AI algorithms integrated into the current beta version of the WHO Skin NTDs App. We will compare the algorithms’ suggested skin conditions with the diagnosis made by a dermatologist in the dermatology department of the Thiès regional hospital center.

The qualitative component aims at exploring the barriers, facilitators, and potential avenues for a larger scale implementation of the WHO Skin NTDs App. To this end, we will collect qualitative data from a range of key stakeholders, including policymakers, leaders and members of leprosy-affected communities, and medical professionals. Results are meant to inform the subsequent refinement of the app and the design and pilot testing of an intervention prototype.

### Study Setting

Skin NTDs are a major public health issue in Senegal, particularly in rural and underserved areas. The key skin NTDs in the country include Buruli ulcer, leprosy, yaws, onchocerciasis, cutaneous leishmaniasis, and scabies, which can lead to long-term disabilities, social stigma, and mental health challenges [1]. In Senegal, the management of NTDs is overseen by the National NTD Control Program, which is under the Directorate of Disease Control within the General Directorate of Public Health of the Ministry of Health and Social Action. To address skin NTDs, a multi-stakeholder approach is employed, which includes nongovernmental actors (eg, Action Damien, Raoul Foundation, DAHW), universities (Thiès, Dakar, Ziguinchor), and various government bodies. The focus of these stakeholders includes disease prevention, medical treatment, research, disability prevention, and rehabilitation.

Compared to other skin NTDs in Senegal, leprosy stands out due to its association with a long-standing isolation policy in the country. Although effective treatment and cure has been available since the 1980s, a law mandating the establishment of 9 social reclassification villages to isolate patients with leprosy was not repealed until 2023 [8,15]. As such, the discriminating character of the previous isolation policy continues to shape leprosy perception and stigma in Senegal. Many former social reclassification villages continue to exhibit active transmission, making them focal points of leprosy prevention activities in Senegal [16-18].

The Thiès regional hospital center, the study site for the quantitative evaluation of the app, is located approximately 70 km from Dakar in the regional capital of Thiès. As of 2023, the health system in the region serves approximately 2,340,000 inhabitants via a network of 335 health huts, 243 health posts, 10 private clinics, and 9 health centers [19]. Of the region’s 5 hospitals, 3 are public and 2 are private. Approximately 70% of the health posts are public [20]. The dermatology department of the Thiès regional hospital center, established in 2002, consists of a consultation room, a treatment room, a secretariat, and a hospitalization unit with a capacity of 9 beds. The department is staffed by 3 dermatologists, who consult with an average of 350 patients per week.

### Quantitative Component: Performance Evaluation of the AI Algorithm

#### Eligibility Criteria

The target population of this component will include any patient seeking treatment for a skin condition at the dermatology department of the Thiès regional hospital center during the study period. This also includes patients referred to the dermatology department from other departments or health facilities. Further inclusion criteria are (1) aged 1 year or older and (2) visible skin lesion on an unidentifiable part of the body. To maintain anonymity of patients, we therefore exclude patients with lesions on the face, fingers, or on or near identifiable scars or tattoos.

#### Sample Size Calculation

Based on the sample size calculation for proportions, we determined that a sample size of 385 images is required to achieve a 95% confidence level with a margin of error of SE 5%, assuming an expected accuracy rate of 75%. In the absence of published performance metrics or internal validation results from the AI algorithm developers, we selected 75% as a conservative estimate of the algorithm’s expected accuracy for the purpose of sample size determination. We conservatively increase our target sample size from 385 to 800 to account for potential exclusions of images due to quality or identifiability concerns and to improve statistical power and robustness.

#### Recruitment and Data Collection

We will collect the images of skin lesions within the dermatology department of the Thiès regional hospital. Using the mobile survey tool Kobo Collect, we will take a picture of the skin lesion from every eligible patient. We will further record the diagnosis made by the dermatologist. We will further collect images of healthy skin from patients, defined as skin being free from dermatological lesions. Data will be collected by a researcher with medical university training. Skin lesion images and dermatologists’ diagnosis will then be linked to the suggested skin conditions from the WHO Skin NTDs App (see Figure 1). All images will be stored on a password-protected server, with access restricted to principal investigators and authorized researchers, ensuring participant data privacy and security.

#### Outcome Measures and Data Analysis

In our primary quantitative analysis, we will first evaluate the performance of the WHO Skin NTDs App’s AI component in detecting whether a skin condition belongs to the broader category of skin NTDs. Specifically, we will assess the Broad Skin Condition Screener, the first of the 2 AI algorithms. We will use the dermatologist’s diagnosis as the reference standard and calculate the following performance metrics:

1. Accuracy: proportion of total cases correctly classified as skin NTDs or skin conditions not caused by NTDs.
2. Sensitivity (recall): proportion of actual skin NTD cases correctly identified by the algorithm (true positive rate).
3. Specificity: proportion of skin conditions not caused by NTDs correctly identified as such (true negative rate).
4. Precision: proportion of cases identified as skin NTDs that are truly skin NTD cases (positive predictive value).
5. *F*_1_-score: harmonic mean of precision and sensitivity, providing a balanced measure of the algorithm’s ability to detect skin NTDs while minimizing false positives.
6. Area under the curve: the algorithm’s ability to distinguish between skin NTD and skin conditions not caused by NTDs, derived from the receiver operating characteristic curve.

We anticipate a degree of class imbalance in our dataset, with skin NTDs being less prevalent than common non-NTD conditions. To mitigate potential bias from this imbalance, we will emphasize the *F*_1_-score. All performance metrics will be reported with 95% CIs, estimated using bootstrap resampling to reflect the uncertainty around our estimates. Our definition of correct prediction is as follows.

1. True positive: an image with any skin NTD is correctly classified as being a skin NTD.
2. True negative: an image without a skin NTD is correctly classified as not being a skin NTD.
3. False positive: an image without a skin NTD is incorrectly classified as being one.
4. False negative: an image with a skin NTD is incorrectly classified as not being one.

We will assess the performance across 3 levels of AI prediction output from the app.

1. Top 1: only the app’s single most likely diagnosis is considered.
2. Top 3: the app presents its 3 most likely diagnoses.
3. Top 5: the app presents its 5 most likely diagnoses.

A prediction will be considered correct if the dermatologist’s diagnosis appeared anywhere within the top 1, top 3, or top 5 predictions. Sensitivity will improve as more predictions are considered, but this comes at the cost of diagnostic precision, as it becomes less clear which suggestion should be acted upon. In our analysis, we will not assess the confidence score or rank the order of the predictions; we will focus solely on whether the correct diagnosis was present within the predicted set.

In cases where the dermatologist provides multiple diagnoses for a lesion (eg, cross-infections), all listed diagnoses will be considered valid labels during evaluation; for instance, if both acne and a skin NTD are noted, the image will be labeled as a skin NTD when assessing the algorithm’s ability to detect skin NTDs.

We will apply the same approach to evaluate the performance of the second algorithm—the Skin NTD Classifier—which is intended to be used only when a skin NTD is suspected based on the output of the first algorithm or the clinician’s judgment. For this analysis, we will include only images diagnosed by the dermatologist as one of the 12 skin NTDs covered by the app, excluding all images of conditions not caused by NTDs. This restriction is necessary because the Skin NTD Classifier algorithm does not offer a “no diagnosis” or “not a skin NTD” option; it always returns one of the 12 disease labels, even if none are appropriate. As a result, specificity cannot be meaningfully assessed unless the input is a confirmed case of one of the 12 target conditions.

In a secondary analysis, we will assess the Skin NTDs classifier algorithm’s performance across the full sample, including both skin lesions and healthy skin. Healthy skin images will be treated as cases with no condition to evaluate the algorithm’s ability to avoid false positives in the absence of disease. We will not include healthy skin images in the evaluation of the second algorithm, which assigns a diagnosis from a fixed list regardless of input. As it does not offer a “no condition” output, specificity cannot be meaningfully assessed in this context.

### Qualitative Component: Feasibility and Acceptability of WHO Skin NTDs App Implementation

#### Eligibility Criteria

The intended populations for this work package are key stakeholders on community, national, and international levels who can provide insights into the feasibility and acceptability of the WHO Skin NTDs App in the Senegalese setting and who can ideate potential use cases of the app, including their respective chances and challenges. These stakeholders, who have to be at least aged 18 years or older and provide written informed consent, will belong to one of the following groups:

1. Policymakers: including representatives of the Ministry of Health, department administrators, and officials on national or local levels involved in decision-making processes for the implementation of health interventions or the treatment of skin diseases in their respective settings.
2. Community leaders: including local and religious leaders, as well as other key stakeholders on community level.
3. Health care providers: including community-level health workers, clinical staff, and doctors working with patients who have skin diseases in the study setting.
4. Patient representatives: including former patients as well as members of patient advocacy groups on local and national levels.
5. Community members: including individuals screened as part of large-scale skin NTD screening efforts.
6. Representatives of nongovernmental organizations and the civil society: including stakeholders involved in the design and deployment of mobile health (mHealth) interventions, in skin disease awareness, diagnosis, and treatment initiatives in Senegal.
7. Software developers: including individuals working on improving this and similar mHealth apps.

#### Sample Size Estimates

The total number of interviews will be based on the principles of saturation (when additional interviews cease to fundamentally provide new themes or new information) [21] and information power [22]. Based on previous experiences in this and similar contexts, at this point in time, we estimate up to 80 interviews across all respondent groups (approximately 40-50 interviews with community members, 10-15 interviews with frontline health workers involved in screening activities and care, and 20 interviews with other stakeholders).

#### Recruitment and Data Collection

Participants will purposively be sampled to ensure a broad range of perspectives and backgrounds and to maximize the depth of information collected [23]. Community members screened as part of larger skin NTD detection efforts will include individuals with and without a skin NTD diagnosis and will be purposively recruited during and after screening campaigns. Local trusted frontline health workers will establish contact to community members and facilitate rapport. Local decision-makers, frontline health workers, and community leaders will be contacted and recruited through their respective office’s official communications channels or through channels established by study team members and partner organizations. If necessary, we will use snowball sampling to identify other potential participants [24].

If contacted individuals are interested in participating, we will provide them with detailed information about the study and schedule a date and time for the interview based on their preferences. Before the interview, we will obtain their informed consent, either in person by signature/fingerprint (if the interview takes place in person) or by digital signature (if the participant prefers the interview to take place via video call).

Qualitative data for this component will be collected via in-depth interviews by using a semistructured interview guide, which will be pilot tested at the start of data collection. Interviews with frontline health workers may also include think-aloud exercises (to further gain practical insights into app engagement) and shared walk components (in which respondents are invited to show locations featuring prominently in their accounts to the interviewer). All interviews will be conducted by trained research assistants in French or Wolof at a place and time of the participant’s choosing and will be audio-recorded. Table 1 summarizes the quantitative and qualitative components of our study.

**Table 1:** Data collection tools to assess the feasibility and potential of the World Health Organization’s (WHO’s) Skin NTDs App to support the detection of cutaneous neglected tropical diseases at the community level in Senegal.

Data collection type	Data collection instrument	Population and recruitment	Sample size (n)	Estimated duration (min)	Timing of data collection
Quantitative	Survey	Patients^a^ Individual^b^ Dermatology department of Thiès hospital^c^ Census^d^ Patient ≥1 year^e^	800	10	Q3 2024 to Q1 2025
Qualitative	In-depth interview guides	Policymakers, community leaders, health care providers, community membersa Individualb Senegal, international (WHO)^c^ Purposived Adult men and women aged ≥18 years who provide written informed consente	~70-80	45-60	Q3 2024 to Q3 2025

^a^Population.

^b^Observation unit.

^c^Sampling unit.

^d^Sampling method.

^e^Eligibility.

#### Data Analysis

Qualitative data analysis will start during data collection via systematic debriefings at regular intervals [25]. Audio recordings of interviews will be transcribed verbatim (potentially employing automated transcription using VINK [from voice to ink; an automated transcription tool] [26], followed by in-depth manual quality checks and corrections) and translated into English. Qualitative data will be analyzed thematically, for example, based on the tenets of the framework approach [25].

Epistemologically, our qualitative component is informed by a constructivist understanding, and we will draw on established guidance and best practices to ensure the quality and rigor of our work, including via the principles set forth by Lincoln and Guba [27], Tracy and Hinrichs [28], and Tracy [29]. We will also triangulate insights across (1) data collectors and analysts representing a range of both emic and etic perspectives (via systematic debriefings, regular team meetings, and collaborative analysis), (2) respondent groups (representing health system supply and demand sides, as well as various decision-making levels), and (3) interviewing techniques (including semistructured interviews, think-aloud exercises, and shared walk components).

In line with this constructivist understanding and the explorative nature of our work, we did not design data collection tools based on specific implementation science frameworks such as the Consolidated Framework for Implementation Research [30] or the RE-AIM (Reach, Effectiveness, Adoption, Implementation, and Maintenance) framework [31]. Similarly, at this point, we do not aim for a purely deductive analytic approach based on predefined framework components. Instead, we will combine inductive and deductive facets: based on aspects that are inductively identified both during data collection and familiarization with the full dataset, as well as in the available literature, we plan to develop and iteratively apply a thematic codebook. The subsequent interpretation of insights from the coded data will then be supported by frameworks, theoretical concepts, and theories as applicable.

### Subsequent Intervention Prototype Development and Pilot Testing

The results from both quantitative and qualitative components are meant to subsequently inform the refinement of the WHO Skin NTDs App and similar tools, as well as the development of an intervention prototype for utilizing the app in the Senegalese context. The exact format of the intervention, timelines, qualitative design data collected over the course of prototype development, as well as pilot testing approaches depend on the outcomes of the performance and feasibility evaluations and will be outlined in future publications.

### Ethical Considerations

This study was approved by the National Ethics Committee for Health Research in Senegal (SEN24/22) and the Ethics Commission of the Medical Faculty Heidelberg (S-132/2024), Germany. No exemptions or waivers were granted. Prior to enrollment, participants will receive detailed verbal and written information about the study. Written informed consent will be obtained from all participants. Participation is voluntary, and participants may opt out of the study at any time without consequence. For secondary analyses, the original informed consent and ethics approvals cover the use of collected data without requiring additional consent. The data will be deidentified before analysis and securely stored in a password-protected file and on a password-protected computer. As the WHO Skin NTDs App itself will not be used to diagnose patients or in the dermatology department, we do not anticipate additional risks or burdens related to false positives or misdiagnoses associated with this study.

## Results

Data collection started in August 2024 and is planned to be completed by September 2025. First quantitative and qualitative results are expected to be submitted for publication by the end of 2025.

## Discussion

This mixed methods study aims to assess the performance of the novel AI component to be integrated in the WHO Skin NTDs App and to explore its potential to support skin disease detection at the community level in Senegal. The performance assessment will be performed in the dermatology department of the regional hospital of Thiès, where we will collect images of skin lesions and the corresponding gold standard diagnosis. We will use these data to assess the performance of the WHO Skin NTD App’s algorithms in detecting skin NTDs and common skin diseases. In the study’s qualitative component, we will conduct in-depth interviews with community, national, and international stakeholders to explore potential use cases of the app to support skin NTD detection in Senegal. The results from these 2 components will subsequently inform the refinement of the app and the development and pilot testing of a community-level intervention.

The availability of mHealth tools using AI image recognition in resource-limited settings is expected to bolster the early detection of skin diseases [8]. Nevertheless, variations in demographic characteristics, skin tones, and the severity and complexity of skin conditions are likely to affect the detection accuracy in real-world application [11,12], but real-world evaluation data remain limited. Our study contributes to filling this gap by providing a performance evaluation combined with the exploration of potential use cases for the WHO Skin NTDs App featuring an AI-based image recognition component.

Although our study, to the best of our knowledge, provides the first comprehensive evaluation of the WHO Skin NTDs App in a francophone context, our performance evaluation will be limited to skin conditions presented at the dermatology department in the regional hospital in Thiès, Senegal. Skin condition prevalence likely differs from that in other regions in Senegal or other countries. Further, the potentially low prevalence of certain skin-NTD conditions in our sample might limit our ability to evaluate the algorithms’ performance on all skin conditions.

We expect our results to enable informed decision-making on the potential use of the WHO Skin NTDs App to support early skin NTD diagnosis in Senegal, particularly in regions with limited access to dermatologists. By providing insights into the potential benefits and risks associated with the app implementation, we aim to inform the development of context-sensitive use cases of the WHO Skin NTDs App and similar tools in Senegal and beyond.

## Appendix

Multimedia Appendix 1 SPIRIT checklist.

## Abbreviations

AI: artificial intelligence
mHealth: mobile health
NTD: neglected tropical disease
RE-AIM: Reach, Effectiveness, Adoption, Implementation, and Maintenance
VINK: from voice to ink
WHO: World Health Organization

## Data Availability

The final dataset used and analyzed for this study will be available from the corresponding author on reasonable request.
